# Heterogeneous distributions in clinical events preceding anticoagulant treatment nonpersistence in patients with venous thromboembolism stratified by active cancer: A nationwide cohort study

**DOI:** 10.1002/cam4.6626

**Published:** 2023-10-26

**Authors:** Dongwon Yoon, Han Eol Jeong, Songhwa Choi, Daye Lee, Ju‐Young Shin, Soo‐Mee Bang

**Affiliations:** ^1^ School of Pharmacy Sungkyunkwan University Suwon South Korea; ^2^ Department of Biohealth Regulatory Science Sungkyunkwan University Suwon South Korea; ^3^ Medical Affairs Pfizer Korea Ltd. Seoul South Korea; ^4^ Samsung Advanced Institute for Health Sciences & Technology Sungkyunkwan University Seoul South Korea; ^5^ Division of Hemato‐Oncology, Department of Internal Medicine, Seoul National University College of Medicine Seoul National University Bundang Hospital Seongnam South Korea

**Keywords:** active cancer, anticoagulant treatment, clinical events, nonpersistence, venous thromboembolism

## Abstract

**Background:**

Nonpersistence in anticoagulation therapy is common and associated with undesirable clinical outcomes in patients with venous thromboembolism (VTE).

**Methods:**

We investigated preceding clinical events of treatment nonpersistence (e.g., switching, discontinuing, or restarting) in VTE patients with and without active cancer using Korean claims database.

**Results:**

Clinically significant events including thromboembolic events, hepatic function change and surgery preceded treatment nonpersistence, but heterogeneous distributions of clinical events were observed in the presence of active cancer. Patients with active cancer had a low rate of clinical events preceding treatment nonpersistence, and new active cancer diagnosis in the nonactive cancer group was most common before the switch to parenteral anticoagulants from warfarin or non‐vitamin K antagonist oral anticoagulants (NOACs).

**Conclusion:**

These findings suggest that clinically significant events can precede treatment nonpersistence and largely paralleled current guidelines for patients with VTE, whereas heterogeneous distributions of clinical events were observed in the presence of active cancer.

## 1 INTRODUCTION

Non‐vitamin K antagonist oral anticoagulants (NOACs) have significantly shifted the therapeutic paradigm in patients with venous thromboembolism (VTE). Recent guidelines recommend NOACs over low molecular weight heparin (LMWH) and vitamin K antagonists in patients with VTE.^1^, ^2^

While guidelines recommend continuing anticoagulant therapy, treatment nonpersistence (i.e., switch, discontinuation, or interruption) is frequent owing to several factors, including patient or physician preferences, costs, and clinical events, such as new‐onset thrombosis, aggravating underlying disease, or adverse events.^3^ Moreover, nonpersistence in the first year affects approximately one in seven patients due to a variety of reasons,^4^, ^5^, ^6^ and this nonpersistence can lead to undesirable clinical outcomes and a substantial disease burden.^7^, ^8^ However, current guidelines provide limited data on the nonpersistence of anticoagulation therapy, particularly in patients with active cancer, as a hypercoagulable state necessitates careful consideration before deciding to continue anticoagulants after clinical events.^9^

Given the potential adverse consequences of nonpersistence in anticoagulant therapy, a more in‐depth understanding of the clinical events preceding nonpersistence in anticoagulant therapy could help optimize their use for VTE. Therefore, we investigated various clinically relevant events before the alteration of anticoagulant treatment, stratified by active cancer, in patients with VTE.

## 2 METHODS

We conducted a population‐based cohort study using Korea's nationwide claims data, the Health Insurance Review and Assessment Service, which provides healthcare‐related information (e.g., sociodemographics, diagnoses, procedures, and prescriptions) for all residents. A validation study found an 82% positive predictive value for diagnoses in claims against electronic medical records.^10^ Between March 2013 and June 2019, we identified all adults (aged ≥18 years) newly diagnosed with VTE and initiated anticoagulant therapy within 30 days of diagnosis. The cohort entry and the index date were the dates of VTE diagnosis and anticoagulant prescription, respectively. Individuals meeting any of the following were excluded: diagnosed with VTE or received anticoagulants within the year before cohort entry; diagnosed with atrial fibrillation/flutter, mechanical heart valve replacement, mitral stenosis, or inferior vena cava filter before the index date; prescribed two or more different oral anticoagulants on the index date; and had a pregnancy record within the 9 months preceding the index date. The cohort study was stratified on active cancer and identified using a unique domestic code for nationwide claims data identification based on a confirmed cancer diagnosis by histopathological, cytological, radiological, and immunological findings within 6 months before the index date.

Index anticoagulant therapy was classified into three discrete groups within each stratified cohort: (1) warfarin (prescribe warfarin alone or within 14 days of parenteral anticoagulants [PAC]); (2) NOAC (prescribe NOACs alone or within 14 days of PACs); and (3) PAC (prescribe unfractionated heparin or LMWH for >14 days). Types of treatment nonpersistence with likely different patterns of clinical events depending on their type were categorized into (1) switch (e.g., prescribe another anticoagulant within 30 days of continuous index anticoagulant treatment), (2) discontinuation (e.g., end of continuous index anticoagulant treatment without switch or re‐initiation), and (3) interruption (e.g., restart index anticoagulant after a gap of no new treatment within 30 days of continuous index anticoagulant treatment).^11^ Clinical events of interest assessed during the 30 days before each nonpersistence included thromboembolic events, major surgery, major bleeding, hepatic or renal function changes, VTE complications, and active cancer. All events had to have occurred for the first time within a 6‐month or 5‐year window for acute (i.e., thromboembolism) or chronic (i.e., cancer) conditions, respectively.^12^ Patients can contribute to multiple events. All analyses were performed using SAS Enterprise Guide 7.1 (SAS Institute Inc., USA).

## 3 RESULTS

We identified 7255 and 48,504 VTE patients with and without active cancer, respectively. Of the new users of anticoagulants, 89.9% (*n* = 6522) of those with active cancer and 93.3% (*n* = 45,240) of those without active cancer did not persist their index anticoagulant during follow‐up. Among these populations, treatment discontinuation was the most common, followed by interruption and switch, regardless of active cancer. In the group without active cancer, 1541 (14.8%), 1273 (4.0%), and 535 (8.2%) patients switched from the warfarin, NOAC, and PAC initiator groups, respectively. Specifically, 1470 (95.4%) patients switched to NOACs from warfarin, 1155 (90.7%) switched to warfarin from NOACs, and 415 (77.6%) switched to NOACs from PACs. Meanwhile, in the group with active cancer, 144 (17.6%), 173 (4.1%), and 208 (9.3%) patients switched to warfarin, NOAC, and PAC therapies, respectively. Among them, 98 (68.1%) patients switched to NOACs from warfarin, 84 (48.6%) switched to PACs from NOACs, and 172 (82.7%) switched to NOACs from PACs.

**FIGURE 1:**
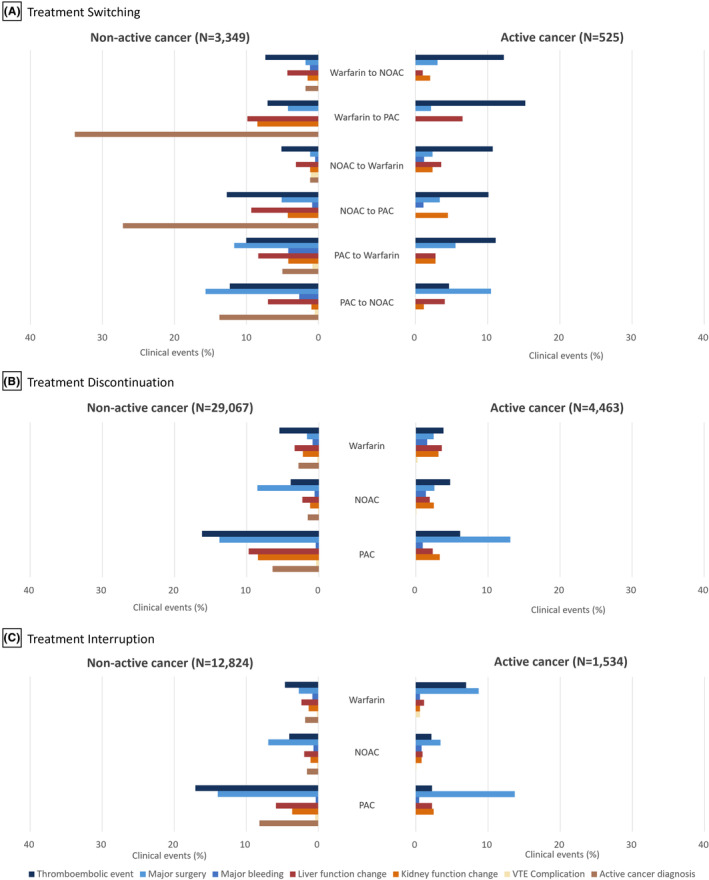
Clinical events preceding treatment nonpersistence in anticoagulant therapy stratified by active cancer, among (A) individuals who switched, (B) those who discontinued, and (C) those who interrupted their anticoagulant therapy. VTE was defined using the following diagnostic codes based on the International Classification of Diseases 10th Revision: I26.0, I26.9, I80.2, and I80.3. Switched anticoagulant needed to last for ≥30 days to be considered a treatment switch, whereas switching to an oral anticoagulant within 2 weeks of initial heparin treatment was not considered a switch given that heparin can be initiated at an acute stage of deep vein thrombosis/pulmonary embolism before the clinical decision to use oral anticoagulants. Thromboembolic event was defined as the composite of pulmonary embolism (among patients with index VTE as deep vein thrombosis only), arterial thromboembolism, ischemic stroke, transient ischemic attack, myocardial infarction, and angina pectoris. Major surgery was defined as the composite of orthopedic, cancer, and benign surgeries. Major bleeding was defined as the composite of intracranial hemorrhage, gastrointestinal bleeding, and other bleeding. Hepatic function change was defined as the new diagnosis of liver disease or abnormal hepatic function. Renal function change was defined as the new diagnosis of chronic kidney disease or acute kidney injury. Complications of VTE were defined as the new diagnosis of post‐thrombotic syndrome and chronic thromboembolic pulmonary hypertension. NOAC, non‐vitamin K antagonist oral anticoagulant; PAC, parenteral anticoagulants; VTE, venous thromboembolism.

Overall, clinical events preceding treatment nonpersistence occurred more frequently in patients without active cancer than in their counterparts across switching, discontinuation, and interruption. The most prevalent clinical event preceding treatment switch among VTE patients without active cancer was thromboembolic events in both the warfarin and NOAC initiators, while major surgery was the most common preceding event in the PAC initiators. Among the warfarin initiators who underwent treatment switch, 7.4% and 7.0% switched to NOACs and PACs, respectively, from warfarin after experiencing a thromboembolic event (Figure 1A). Moreover, 5.1% and 12.7% of the NOAC initiators switched to warfarin or PAC, respectively, after encountering a thromboembolic event. In the PAC initiators, 11.7% and 15.7% of patients switched to warfarin and NOAC, respectively, after experiencing major surgery (Figure 1A). A new active cancer diagnosis in the nonactive cancer group was the most common before the switch to PACs from warfarin (34%) or NOACs (27%; Figure 1A).

Before treatment discontinuation, a thromboembolic event was the most frequent clinical event in VTE patients without active cancer for the PAC (16.2%) and warfarin (5.5%) initiators, whereas major surgery was the most common preceding event for the NOAC‐based therapy group (8.5%). For VTE patients with active cancer, thromboembolic events were common preceding events for warfarin (3.8%) and NOAC (4.8%) initiators, but major surgery was the most prevalent preceding event for the PAC initiators (13.1%) (Figure 1B).

The results for treatment interruption were analogous to those of discontinuation in both groups. However, in VTE patients with active cancer, major surgery was the most common preceding event for treatment interruption in the PAC‐only group (13.7%), warfarin‐based therapy group (8.7%), and NOAC‐based therapy group (3.4%) (Figure 1C).

## 4 DISCUSSION

In this large‐scale nationwide cohort study, we observed that the most frequent clinical events preceding anticoagulant treatment changes (such as switching or discontinuation) among VTE patients were thromboembolic events and major surgery, regardless of active cancer.

Overall, patients with active cancer had a low rate of clinical events preceding treatment nonpersistence, likely owing to the lack of clear recommendations for these patients. Furthermore, clinicians' decisions not to take action after such events to address the hypercoagulable state cannot be ruled out.^13^ Additionally, the heterogeneous hemodynamic status in the presence of active cancer needs to be considered regarding treatment nonpersistence. Moreover, an incident cancer diagnosis may have triggered switches to PACs in patients without active cancer, as previous guidelines recommended PACs for patients with active cancer.^14^, ^15^ Switching from PACs to NOACs was prevalent because NOACs are an alternative for initial treatment in patients with active cancer.^1^

Thromboembolic events are known to have a higher incidence and mortality in patients with VTE compared to the general population, and are particularly significant events that warrant urgent care in patients receiving anticoagulants.^16^, ^17^ Our findings support this because thromboembolic events were common preceding treatment nonpersistence, suggesting that patients probably received urgent care with consideration for their hemodynamic status upon experiencing such events.

Hepatic function changes were another notable clinical event before treatment nonpersistence. As conventional clinical trials have generally excluded patients with severe hepatic dysfunction, evidence on the safety and efficacy of NOACs in these patients is limited. Consequently, PAC is often the preferred treatment for patients with liver disease.^18^ Our study's findings support this by showing consistent patterns of switching to PAC from warfarin or NOACs in patients with liver disease.

Perioperative management of anticoagulant treatment requires a careful balance of the risk of bleeding and thrombosis. Consistent with current recommendations for perioperative management of anticoagulation, which suggest discontinuing or interrupting therapy before surgeries, major surgery was prevalent preceding treatment nonpersistence.^19^, ^20^

The study strengths include using nationwide data to identify all eligible patients with VTE and a special domestic code to accurately define active cancer. However, the study also has limitations. First, the possible outcome misclassification given the use of diagnosis or procedural codes to capture clinical events. Second, the absence of laboratory data (i.e., international normalized ratio). Lastly, the causal relationship could not be verified due to the nature of the claims data, however, the study could identify the temporal relationship between clinical events and treatment nonpersistence. Further research using electronic medical records could provide a better understanding of the causal and temporal associations between clinical events and nonpersistence in anticoagulant treatment.

## 5 CONCLUSIONS

Clinically significant events can precede treatment nonpersistence, whereas heterogeneous distributions of clinical events were observed in the presence of active cancer. These findings offer valuable insights for clinicians and patients with VTE, guiding clinical decision making. Additionally, they contribute novel data on Asian population, addressing existing knowledge gaps primarily based on evidence from Western populations.

## AUTHOR CONTRIBUTIONS

**Dongwon Yoon:** Conceptualization (lead); data curation (equal); formal analysis (equal); investigation (equal); methodology (equal); writing – original draft (lead); writing – review and editing (equal). **Han Eol Jeong:** Data curation (equal); formal analysis (equal); investigation (equal); methodology (equal); writing – review and editing (equal). **Songhwa Choi:** Supervision (equal); writing – review and editing (equal). **Daye Lee:** Project administration (equal). **Ju‐Young Shin:** Data curation (equal); formal analysis (equal); investigation (equal); methodology (equal); writing – review and editing (equal). **Soo‐Mee Bang:** Conceptualization (equal); investigation (equal); supervision (equal); writing – original draft (equal); writing – review and editing (equal).

## FUNDING INFORMATION

This study was sponsored by Pfizer and Bristol‐Myers Squibb.

## CONFLICT OF INTEREST STATEMENT

The authors Ju‐Young Shin and Soo‐Mee Bang received honoraria as speakers and/or consultants from Pfizer and Bristol Myers Squibb. Songhwa Choi is a previous employee of Pfizer Korea and Daye Lee is an employee of Pfizer.

## ETHICS STATEMENT

The Sungkyunkwan University Bioethics Committee (IRB No: SKKU 2020–12‐010).

## Data Availability

The data analyzed in this study was obtained from the Korea Health Insurance Review & Assessment Service (HIRA) claims database, the following licenses/restrictions apply: requests to access these datasets must first be approved by the HIRA Service. Requests to access these datasets should be directed to the HIRA Service, opendata.hira.or.kr.
